# Development and feasibility testing of a new device for home-based leg heat therapy in patients with lower extremity peripheral artery disease

**DOI:** 10.1016/j.jvscit.2024.101676

**Published:** 2024-11-08

**Authors:** Bohyun Ro, John P. Spence, Paul A. Spence, Christian Buckley, Raghu L. Motaganahalli, Bruno T. Roseguini

**Affiliations:** a Department of Health and Kinesiology, Purdue University, West Lafayette, IN; b Aquilo Sports LLC, Louisville, KY; c Division of Vascular Surgery, Department of Surgery, Indiana University School of Medicine, Indianapolis, IN

**Keywords:** Peripheral artery disease, Heat therapy, Mobility

## Abstract

People with symptomatic lower extremity peripheral artery disease (PAD) suffer from severe leg pain, walking impairment, and reduced quality of life, but few effective treatments are available. Emerging evidence suggests that regular heat therapy (HT) may improve cardiovascular and physical function in patients with PAD. However, the lack of accessible, practical modalities for unsupervised HT, especially for elderly individuals, has hindered clinical implementation. The goals of this study were to design and assess the feasibility of a portable leg HT system for elderly patients with PAD. Building on a cryotherapy water-circulating device used in sports recovery, we developed a new prototype system consisting of a single-touch controller unit integrating a heater, water pump, and air pump, and leg sleeves with inner-layer water-circulating pads and an outer layer of inflatable bladders. The system was designed to ensure efficient heat transfer through gentle pneumatic inflation, adapting to varying limb dimensions. Safety features included temperature sensors with auto shut-off and a built-in timer. The prototype's feasibility and safety were evaluated in a single-arm pilot trial with six symptomatic patients with PAD, who were asked to apply the therapy daily for 90 minutes for 12 weeks. Primary outcomes included completion rates, safety, and device usability. Secondary outcomes were changes in blood pressure, 6-minute walk distance, calf strength, sit-to-stand performance, and quality of life. Participants underwent a 90-minute supervised treatment session with the prototype HT units to assess the acute physiological responses before starting the 12-week intervention. Leg HT gradually increased leg skin temperature from 33.8 ± 0.8°C to 38.7 ± 0.7°C at 90 minutes and reduced arterial blood pressure, with mean reductions of 13 mm Hg in systolic and 12 mm Hg in diastolic blood pressure after treatment. All participants completed the 12-week program without serious adverse events, indicating that leg HT is safe and well-tolerated. The 6-minute walk distance improved by an average of 32 m, coupled with increased calf muscle strength and reduced time for the sit-to-stand test. Improvements were also observed in self-reported walking speed and quality of life. This study represents the first step in developing a portable leg heating system for elderly patients with PAD, demonstrating that home-based leg HT is feasible and safe. However, further engineering refinements are needed to enhance portability, simplify application, and encourage long-term adherence. Developing methods to track compliance with the treatment regimen will be crucial for the success of this unsupervised, home-based therapy.

Lower extremity peripheral artery disease (PAD), a manifestation of systemic atherosclerosis, affects >236 million individuals worldwide.^1^ Approximately 1 in 10 individuals aged 70 years and nearly 1 in 5 people >80 years have PAD.^1^ Patients with PAD experience a lower quality of life compared with their healthy counterparts, largely due to to a significant decline in physical functioning.^2^ Severe exercise intolerance in these patients limits their capacity for daily physical activity and accelerates mobility loss.^3^^,^^4^ Supervised exercise training is the most effective therapy for improving functional status and reducing leg symptoms.^5^ However, supervised exercise training is prescribed rarely,^6^ and very few structured programs are available.^7^ Home-based exercise programs are a potential alternative, but face significant challenges. Simply advising patients to walk or offering remote support via activity monitors and telephone coaching are often ineffective.^8^^,^^9^ Success typically requires frequent visits to meet with a coach, which can be impractical for many.^10^ Additionally, poor physical health and persistent leg pain during exercise make exercise regimens difficult or unappealing for numerous patients.^11^

Regular exposure to heat therapy (HT), particularly through sauna and hot tub treatments, has emerged as a promising approach to alleviate the symptoms of PAD and improve functional performance.^12^, ^13^, ^14^ Tei et al^13^ first reported a case where a patient with chronic limb-threatening ischemia experienced complete healing of a large skin ulcer after 15 weeks of far-infrared dry sauna therapy, thereby avoiding limb amputation. Daily sessions of far-infrared dry sauna for 6 to 10 weeks improved leg pain, enhanced 6-minute walking distance, and increased ankle-brachial index (ABI) in patients with PAD.^12^^,^^14^ A single 30-minute session of waist-level hot water immersion has been shown to cause a three-fold increase in blood flow in the popliteal artery and reduce the mean arterial blood pressure by 22 mm Hg in patients with PAD.^15^ Additionally, hot water immersion followed by calisthenics 3 to 5 days per week over 12 weeks has been shown to improve walking distance and resting blood pressure in patients with PAD.^16^

The clinical implementation of HT has been hampered by the lack of accessible, easily applicable modalities. Saunas and hot water immersion are largely unavailable, costly, and often require patients to travel to a facility for treatment. Although heat packs, electric heating pads, and electric blankets are widely available, they carry a high risk of contact burns, especially in patients with peripheral neuropathy, which is commonly associated with diabetes mellitus and PAD.^17^ Water-circulating pads may be superior to electric heating pads and thermal blankets owing to their even heat distribution, precise temperature control, and enhanced safety. A recent study on patients with knee osteoarthritis revealed that most patients preferred treatment with a water-circulating garment system over a heating pad.^18^ A garment originally developed for thermal management of military crews, consisting of a network of polyvinyl chloride tubing sewn onto tight-fitting elastic fabric, was recently repurposed to evaluate the acute responses to leg heating in patients with PAD. Exposure to leg HT for 90 minutes using water-circulating trousers resulted in a significant decrease in blood pressure and a substantial increase in popliteal artery blood flow.^19^ A subsequent pilot trial demonstrated that unsupervised, home-based leg HT using this garment system is safe, well-tolerated, and elicits meaningful changes in walking ability in patients with symptomatic PAD.^20^ The water-circulating garment used in the aforementioned trial was not designed specifically for rehabilitation and had several drawbacks, such as limited sizing, poor skin contact, and coverage over only a limited surface area. There are no commercially available water-circulating systems for HT that cover the entire lower body and are suitable for elderly individuals with limited mobility.

Accordingly, the primary goal of this study was to design a clinical-grade, easy-to-use leg HT system for elderly individuals suffering from PAD. Building on an existing cryotherapy water-circulating device designed for sports recovery, we developed a new prototype system consisting of leg sleeves composed of an inner layer of polyurethane water-circulating pads and an outer layer of six inflatable bladders. The feasibility and safety of the prototype units were evaluated in a single-arm pilot trial involving six patients with symptomatic PAD. The primary outcomes were completion rates, safety, and device usability. Secondary outcomes included changes in blood pressure, 6-minute walk distance, calf strength, performance on the five times sit-to-stand test, and quality of life as measured by the Walking Impairment Questionnaire (WIQ).

## Methods

### Device design

**Fig 1:**
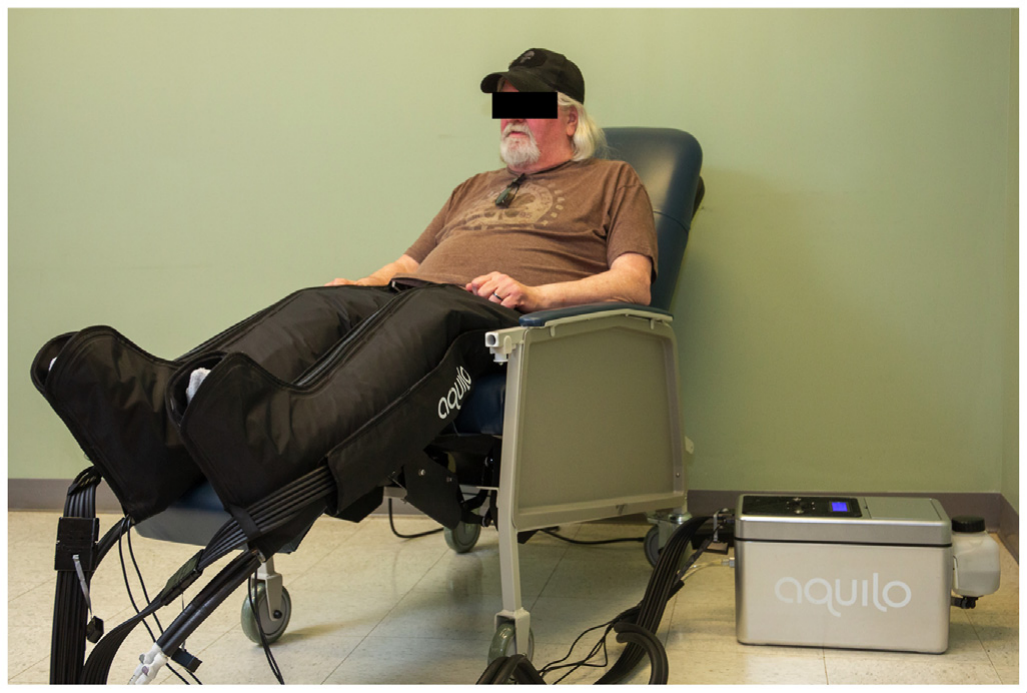
The Aquilo leg heat therapy (HT) prototype system is designed for home-based use by elderly individuals suffering from symptomatic lower extremity peripheral artery disease (PAD). The system features a control unit that houses the user interface, electronic controllers, water and air pumps, and a water reservoir. It is connected to leg sleeves that contain water-circulating pads surrounded by inflatable air bladders. The system heats water to 41°C, which is then circulated through the pads. The air bladders intermittently inflate to 15 mm Hg, ensuring consistent contact between the water-circulating pads and the skin. The treatment is preprogrammed to run for 90 minutes.

**Fig 2:**
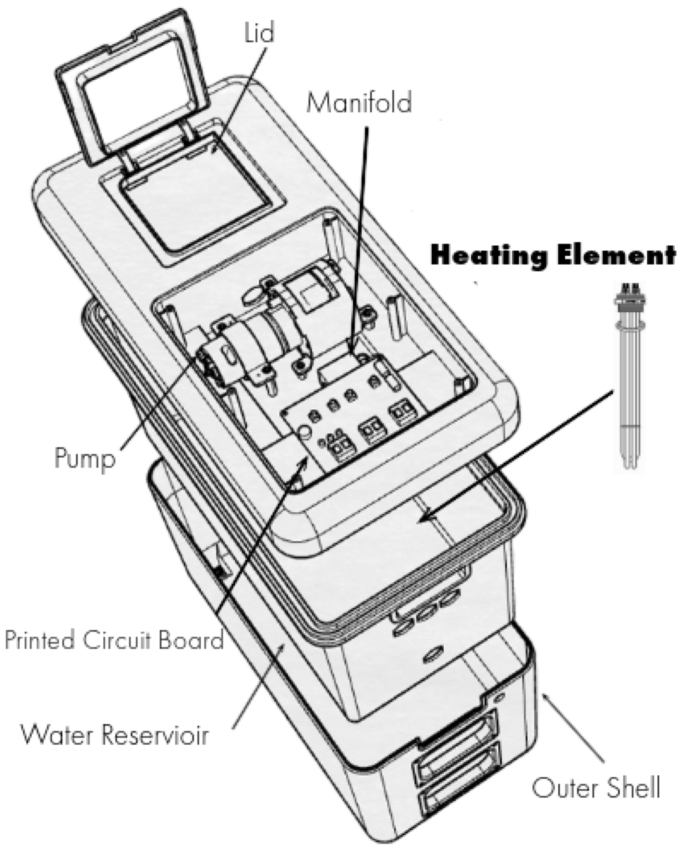
The control unit was redesigned and reengineered from an existing cryotherapy device initially used for sports recovery. The original system lacked temperature control, relying solely on ice to cool the water. Several key modifications were made to enhance the system, including the integration of a heater into the reservoir for controlled heating and the addition of software and hardware to program air pressure, enabling gentle inflation of the six bladders surrounding the thermal layer. A new user interface was developed, featuring a single power button to turn the device on and begin heating the water, along with a start button to activate the water and air pumps for treatment and initiate a 90-minute timer. Temperature monitoring was also implemented both in the reservoir and on the leg, with an automatic shutoff set for temperatures exceeding 42°C. Additionally, a reservoir water level sensor and alarm were installed to ensure proper operation.

**Fig 3:**
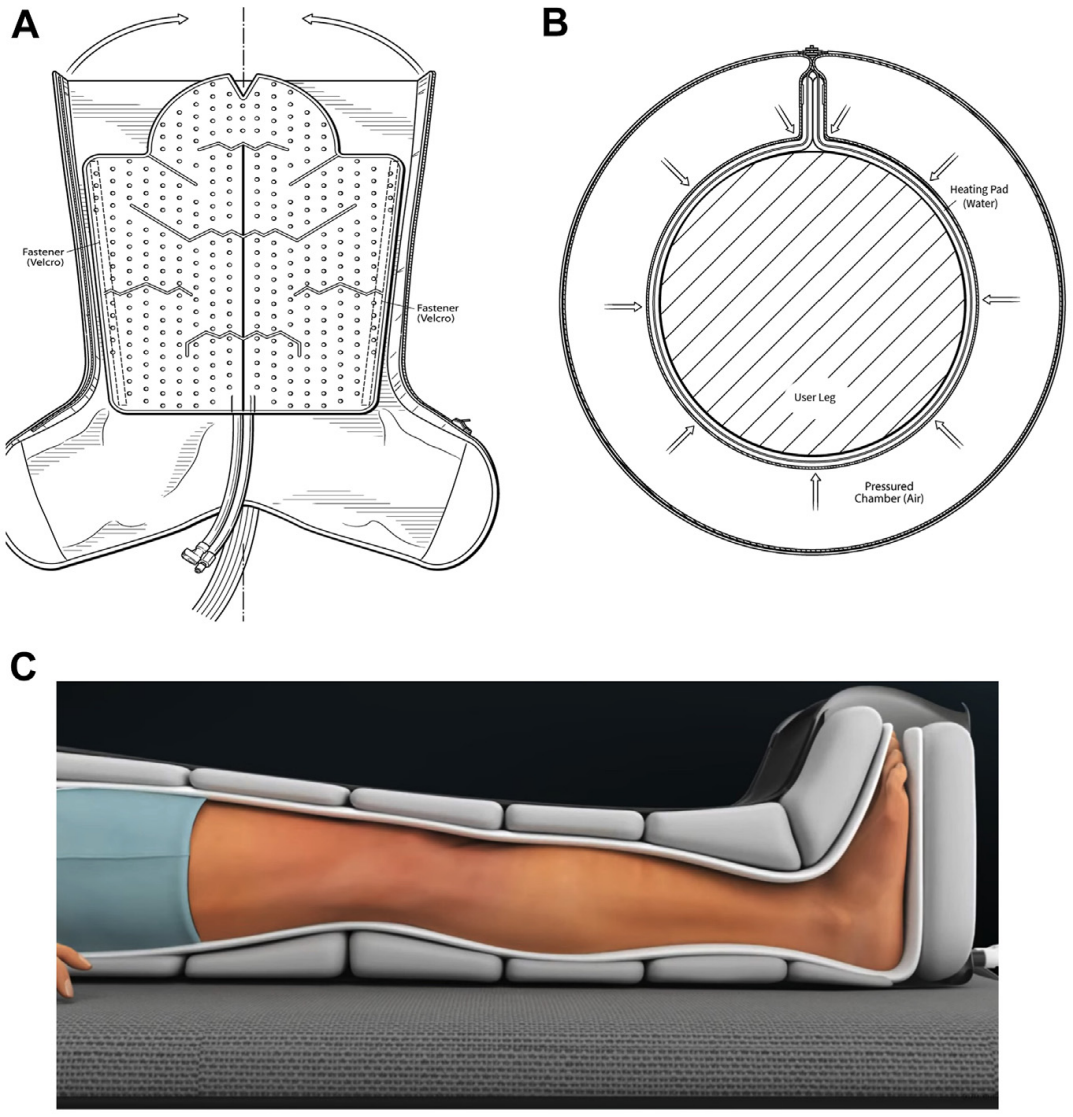
The leg sleeves consist of a large water-circulating pad encircled by six inflatable bladders. The system is programmed to gently inflate the bladders to 15 mm Hg over approximately 10 to 15 seconds. This pressure is maintained for an additional 15 seconds before being reduced to 0 mm Hg. The sleeves are equipped with thermistors to monitor skin temperature continuously, and the system is programmed to automatically shut off if the temperature exceeds 42°C.

Fig 1, Fig 2, Fig 3 provide an overview of the prototype leg HT system, adapted from a cold compression device for sports recovery. The system consists of a control unit, tubing, and leg sleeves (Fig 1). The control unit houses the user interface, electronic controllers, water and air pumps, and a water reservoir (Fig 2). Each leg sleeve contains six inflatable chambers surrounding a water-circulating pad (Fig 3). Operation begins by activating the water heater and circulator, gradually heating the water to 41°C over 40 minutes. Once at temperature, the user initiates treatment, triggering the air pump to inflate the six bladders to 15 mm Hg over 15 seconds. The pressure is then held for another 15 seconds before being reduced to 0 mm Hg at the 30-second mark. This cycle repeats for 90 minutes, after which the system shuts off automatically, prompting sleeve removal. Safety features include a 4 × 20 LCD screen for instructions and status updates, and software that maintains target temperature while activating a safety mode if temperatures exceed 42°C. The system is equipped with thermocouples for continuous skin temperature monitoring, a float sensor to maintain water levels, and safeguards to detect and respond to sensor errors, ensuring safe operation throughout the treatment.

### Trial overview

Device feasibility, safety, and usability were evaluated in an open-label, single-arm pilot study performed at Purdue University in West Lafayette, Indiana. Participants were provided the prototype HT device and were asked to apply the therapy daily (90 minutes, 7 days/week) for 12 weeks. The first participant was enrolled on May 30, 2023. The final follow-up occurred on February 7, 2024. The protocol was approved by Purdue's Institutional Review Board (2022-375), and registered with the United States Library of Medicine on clinicaltrials.gov (NCT05335161). Written, informed consent was obtained, and all procedures adhered to the requirements of the US Federal Policy for the Protection of Human Subjects (45 CFR, Part 46), and support the general ethical principles of the Declaration of Helsinki. Data supporting the findings of this study are available upon reasonable request.

### Participants

Patients with symptomatic PAD were recruited through flyers, online advertisements, and referrals from local peripheral vascular disease clinics. Men and women 60 years or older with a resting ABI of ≤0.90 in at least one leg were included in the study. Individuals with a resting ABI of >0.91 at baseline were eligible if their ABI decreased by ≥20% after a heel-rise test. The exclusion criteria included the presence of critical limb ischemia (ischemic rest pain or ischemia-related, nonhealing wounds, or tissue loss), prior leg amputation, exercise tolerance limited by factors other than leg pain (eg, angina, arthritis, severe lung disease, etc), recent lower extremity revascularization or orthopedic surgery, use of a walking aid other than a cane, active cancer, chronic kidney disease (estimated glomerular filtration rate of <30), inability to fit into water-circulating trousers, a Mini-Mental Status Examination score of <23, and impaired thermal sensation in the leg.

### Experimental protocol

Participants attended two baseline laboratory visits. Before each session, they were instructed to fast overnight, avoid exercise for 24 hours, and refrain from smoking for 4 hours. During the first visit, participants provided informed consent, completed a medical history form, had their leg symptoms assessed using the San Diego Claudication Questionnaire, completed the Mini-Mental Status Examination, and underwent a thermal sensation test with heating pads on their thighs and calves. The ABI was assessed following American Heart Association guidelines. After a 10-minute rest, a handheld Doppler ultrasound probe was used to measure systolic pressures in the posterior tibial, dorsalis pedis, and brachial arteries. The ABI for each leg was calculated by dividing the average dorsalis pedis and posterior tibial pressures by the average of the brachial pressures. Participants were then familiarized with the 6-minute walk test, isokinetic calf strength testing, and the five-times sit-to-stand test.

The second visit, conducted ≥48 hours later, included (1) blood pressure measurement, (2) a 6-minute walk test, (3) torque assessments of the plantar flexors using an isokinetic dynamometer, (4) the five-times sit-to-stand test, and (5) quality of life assessment via the WIQ. After baseline tests, participants underwent a supervised 90-minute treatment session with the prototype HT device to monitor acute changes in skin temperature and blood pressure. Thermocouples (MLT422; AD Instruments, Colorado Springs, CO) were taped to the calf and thigh to measure leg skin temperature, and systolic and diastolic blood pressures were recorded every 5 minutes.

Participants were given the prototype device for daily use while lying down or seated with legs extended. They logged therapy sessions and measured blood pressure before and after each session using a portable monitor (Omron 3 Series Upper arm, Omron Healthcare Co. Ltd., Kyoto, Japan). Weekly check-ins with the study coordinator tracked therapy dates, times, blood pressure readings, and any adverse effects. Outcomes were reassessed at the end of the 12-week intervention, with participants stopping treatment 48 hours before the final evaluations to isolate chronic effects.

### Outcome measures

#### Feasibility end points (primary outcomes)

##### Completion rate

The completion rate was assessed by tracking the number of participants who completed the study protocol within the specified timeframe relative to the number of participants enrolled in the study.

##### Safety

Safety was assessed by monitoring the occurrence of adverse events. The research coordinator called patients weekly to track any adverse events throughout the intervention.

##### Device usability

Participants were asked to grade the device usability at the end of the experiment using the System Usability Scale. Psychometric properties of the System Usability Scale have been demonstrated, including reliability and sensitivity.^21^

### Secondary outcomes

#### 6-Minute walk test

Following the guidelines from the American Thoracic Society,^22^ participants received standardized instructions and were asked to walk the greatest distance possible by walking back and forth along a 100-ft corridor for 6 minutes. Performance on this corridor-based test is associated with physical activity levels during daily life in people with PAD.^23^

#### Blood pressure

Blood pressure measurement was conducted using an automated device (Tango+, Suntech Medical, Morrisville, NC), following the recommendations of the American Heart Association.^24^ Participants were asked to sit in a chair with their feet flat on the floor and their back supported, remaining seated for 5 minutes without talking or moving before the first blood pressure reading was taken. Clothing covering the location of cuff placement was removed to ensure accurate readings, and the participant's arm was supported, resting on a desk. At the first visit, blood pressure was recorded in both arms, and the arm that gave the higher reading was used for subsequent measurements.

#### Calf strength

Plantar flexor strength and endurance are lower in people with PAD when compared with individuals without PAD.^25^^,^^26^ Importantly, poorer plantar flexion strength and leg power are associated with higher all-cause mortality among people with PAD.^27^ Torque assessments of the plantar flexors of the most symptomatic leg or the leg with the lowest ABI were conducted using an isokinetic dynamometer (Biodex System 4 Pro, Biodex Medical Systems, Shirley, NY). The participants were positioned supine on the dynamometer chair with their knees flexed at 90°. Before maximal testing, they completed a standardized warm-up consisting of three submaximal contractions, each held for 5 seconds at approximately 50% of perceived maximal effort. Following the warm-up, participants performed three maximal voluntary isometric contractions, each lasting 5 seconds, with a 60-second rest period between attempts to minimize fatigue. Standardized verbal encouragement was provided during each maximal voluntary isometric contraction to ensure maximal effort. The trial that produced the highest peak torque value (in N·m) was selected for analysis.

#### The Five Times Sit to Stand Test

Participants were asked to sit in a chair with arms folded across their chest and stand five times consecutively as quickly as possible. The time it took to complete five chair rises was measured.^28^^,^^29^

#### WIQ

Patient-reported perceptions of walking ability was assessed using the WIQ.^30^ This disease-specific questionnaire assesses the ability of patients with PAD to walk defined distances and speeds and to climb stairs, with scores ranging from 0 to 100.^30^ Higher scores reflect better community-based walking ability.^31^

### Statistical analysis

This small pilot trial aimed to test prototype HT devices and assess their safety and feasibility in patients with PAD. The primary objective was to collect initial data to refine the device design and engineering and to guide the development of future large-scale studies.^32^ Given the exploratory nature of this trial, no power analysis was conducted.^32^ Changes in each outcome from baseline to the 12-week follow-up were compared using paired two-sample *t* tests. A two-sided *P* value of <.05 was considered statistically significant. Before the onset of the intervention, participants underwent a supervised HT session, during which skin temperature and blood pressure were monitored every 5 minutes for the entire 90-minute session. Skin temperature and blood pressure responses during this session were analyzed using repeated-measures analysis of variance. When a significant main effect was detected, Sidak multiple comparisons tests were used to assess differences between time points relative to baseline. A *P* value of <.0028 was considered statistically significant for the Sidak-adjusted comparisons. All statistical analyses were performed using Prism 10 (Version 10.3.0 (461), July 26, 2024).

## Results

**Fig 4:**
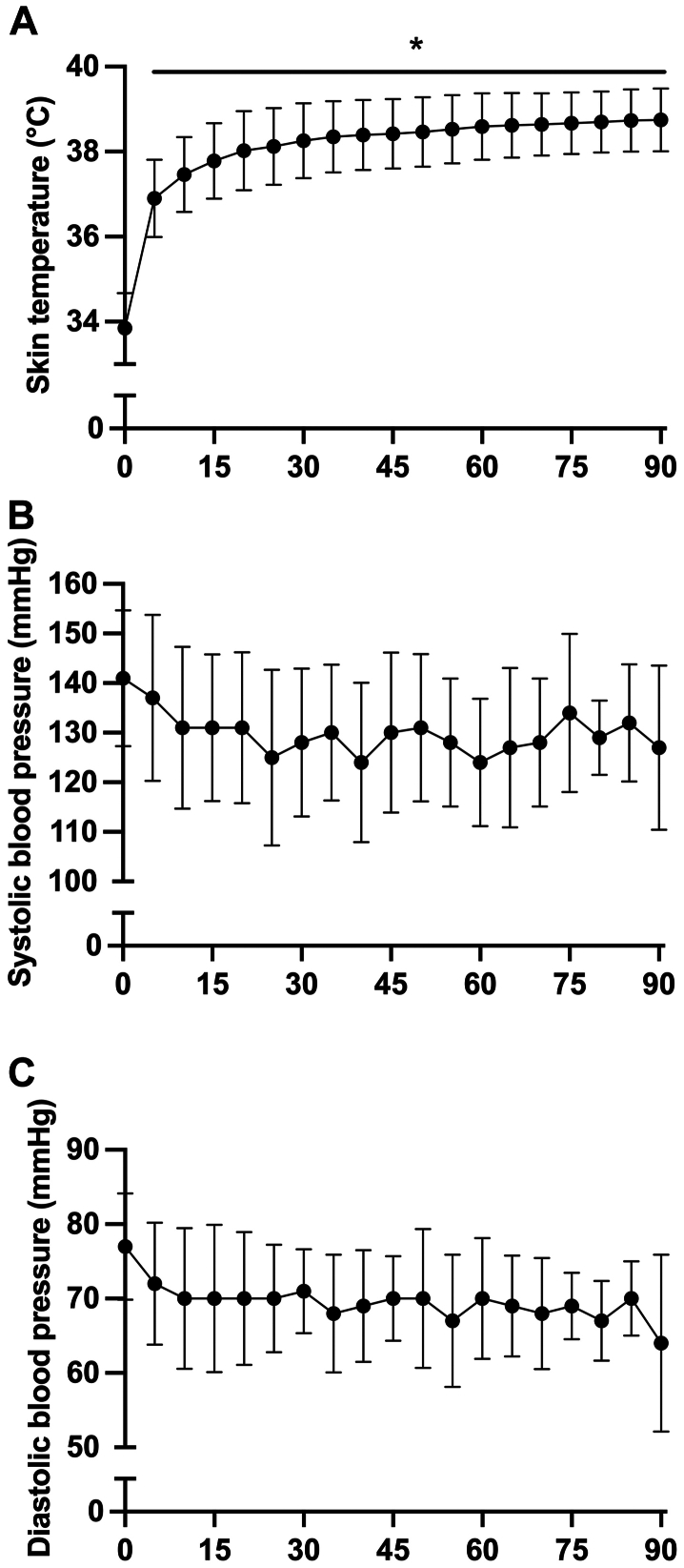
Average leg skin temperature **(A)** and blood pressure changes **(B** and **C)** during a supervised 90-minute leg heat therapy (HT) session using the prototype units. Data were analyzed using repeated-measures analysis of variance. When a significant main effect was detected, Sidak adjustment was applied to compare the time points relative to the baseline. ∗*P* < .028 vs time 0 (baseline).

The demographic and clinical profiles of the patients with PAD are shown in Table I. All six participants reported leg symptoms during walking, with three experiencing thigh pain, one reporting both thigh and buttock pain, and three reporting calf pain. Before commencing the 12-week intervention, participants underwent a supervised 90-minute treatment session with the prototype HT units to assess the acute physiological responses to the treatment. The average changes in skin temperature and blood pressure during the session are displayed in Fig 4. Exposure to HT induced a gradual increase in leg skin temperature from 33.8 ± 0.8°C to 38.7 ± 0.7°C at 90 minutes, F(1.838, 9.190) = 153.2, *P* < .0001 (Fig 4, *A*). HT also promoted a reduction, albeit not statistically significant, in arterial blood pressure. The mean decrease in systolic and diastolic pressures after 90 minutes of heat treatment was 13 mm Hg and 12 mm Hg, respectively (Figs 4, *B* and *C*).

**Table I:** Baseline characteristics of the participants

Variables	All participants
No.	6
Age, years	70.50 ± 7.45
Sex	
Male	4 (66.67)
Female	2 (33.33)
Race	
White	6 (100.00)
Height, cm	171.78 ± 12.15
Weight, kg	86.53 ± 20.94
BMI, kg/m^2^	29.10 ± 5.29
ABI	
Most affected leg	0.65 ± 0.20
Other leg	0.83 ± 0.06
Type 2 diabetes	
No	2 (33.33)
Yes	4 (66.67)
Hypertension	
No	2 (33.33)
Yes	4 (66.67)
MMSE	29.50 ± 1.22
Blood pressure, mm Hg	
Systolic blood pressure	131.06 ± 9.41
Diastolic blood pressure	68.11 ± 6.98
Medication	
Statin	5 (83.33)
Antidiabetic	4 (66.67)
Antihypertension	6 (100.00)
Anti-inflammatory	5 (83.33)
6-Minute walk distance, m	389.21 ± 75.45
Calf strength, Nm	32.12 ± 17.98
FTSTS, s	10.40 ± 4.20

*ABI,* Ankle-brachial index; *BMI,* body mass index; *FTSTS,* five times sit to stand test; *MMSE,* mini-mental status examination.

Values are mean ± SD or number of participants (%).

All participants completed the 12-week program, and no serious adverse events were observed, confirming previous findings that leg HT is safe and well-tolerated in symptomatic PAD.^20^ One participant experienced discomfort in the foot and ankle while applying the therapy. The participant was advised to protect the affected areas and completed the 12-week intervention without complications. Participants reported completing more than 90% of required home-based treatment sessions. The responses to the product usability questionnaire are shown in Table II.

**Table II:** Patient-reported device usability as assessed by the system usability scale

System Usability Scale questionnaire	Answer
1. I think that I would like to use this product frequently.	2.00 ± 1.41
2. I found the product unnecessarily complex.	0.50 ± 0.55
3. I thought the product was easy to use.	1.83 ± 1.72
4. I think that I would need the support of a technical person to be able to use this product.	0.67 ± 1.63
5. I found the various functions in the product were well integrated.	1.67 ± 1.51
6. I thought there was too much inconsistency in this product.	0.50 ± 0.84
7. I imagine that most people would learn to use this product very quickly.	2.50 ± 1.38
8. I found the product very awkward to use.	2.00 ± 1.26
9. I felt very confident using the product.	2.50 ± 0.84
10. I needed to learn a lot of things before I could get going with this product.	0.33 ± 0.52
Total	66.25 ± 11.59

Values are means ± standard deviation. To score the system usability scale in this study, participants completed a 10-item questionnaire with 5 response options each. For odd-numbered items, 1 point was subtracted from the user's response. For even-numbered items, the user's response was subtracted from 5. This conversion scales all responses to a range of 0 to 4, with 4 representing the most positive response. The converted scores for each item were then summed, and the total was multiplied by 2.5 to convert the possible score range from 0 to 100, providing an overall usability score.

**Fig 5:**
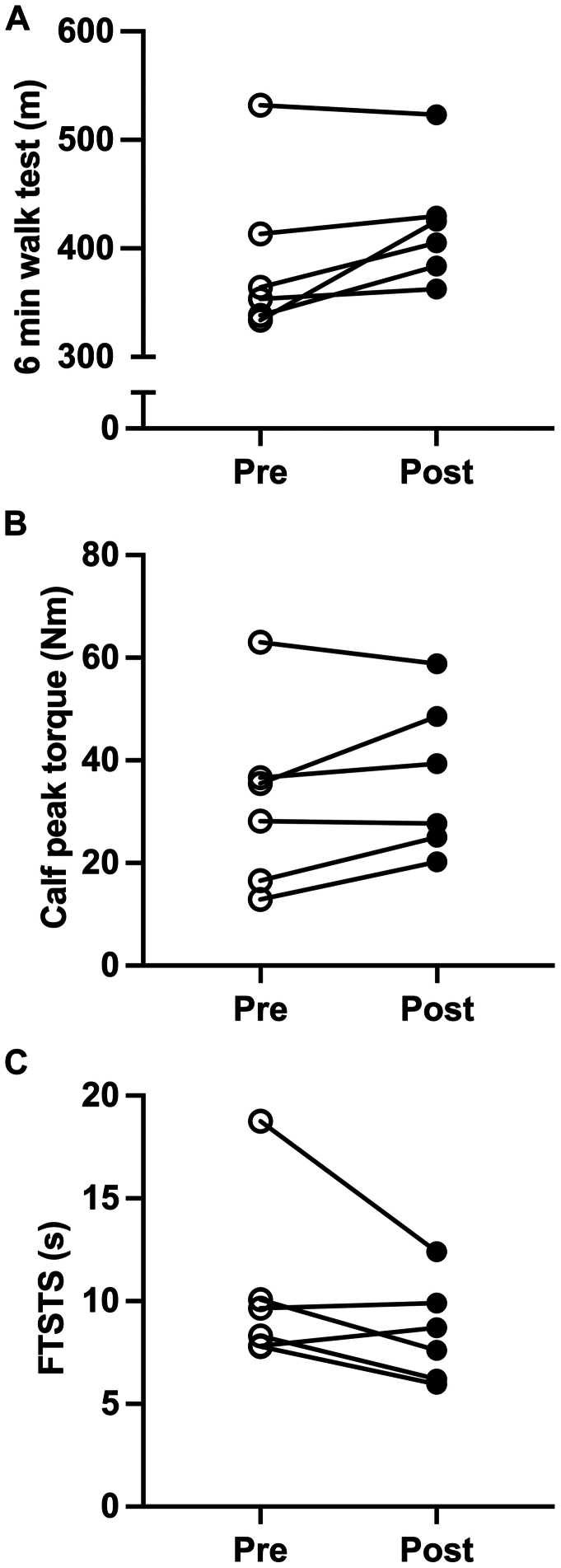
Individual responses to the 6-minute walk test **(A)**, peak torque of the plantar flexors assessed using isokinetic dynamometry **(B)**, and the Five Times Sit to Stand Test **(C)** before and after the 12-week home-based intervention. Data were compared using paired two-sample *t* tests.

**Fig 6:**
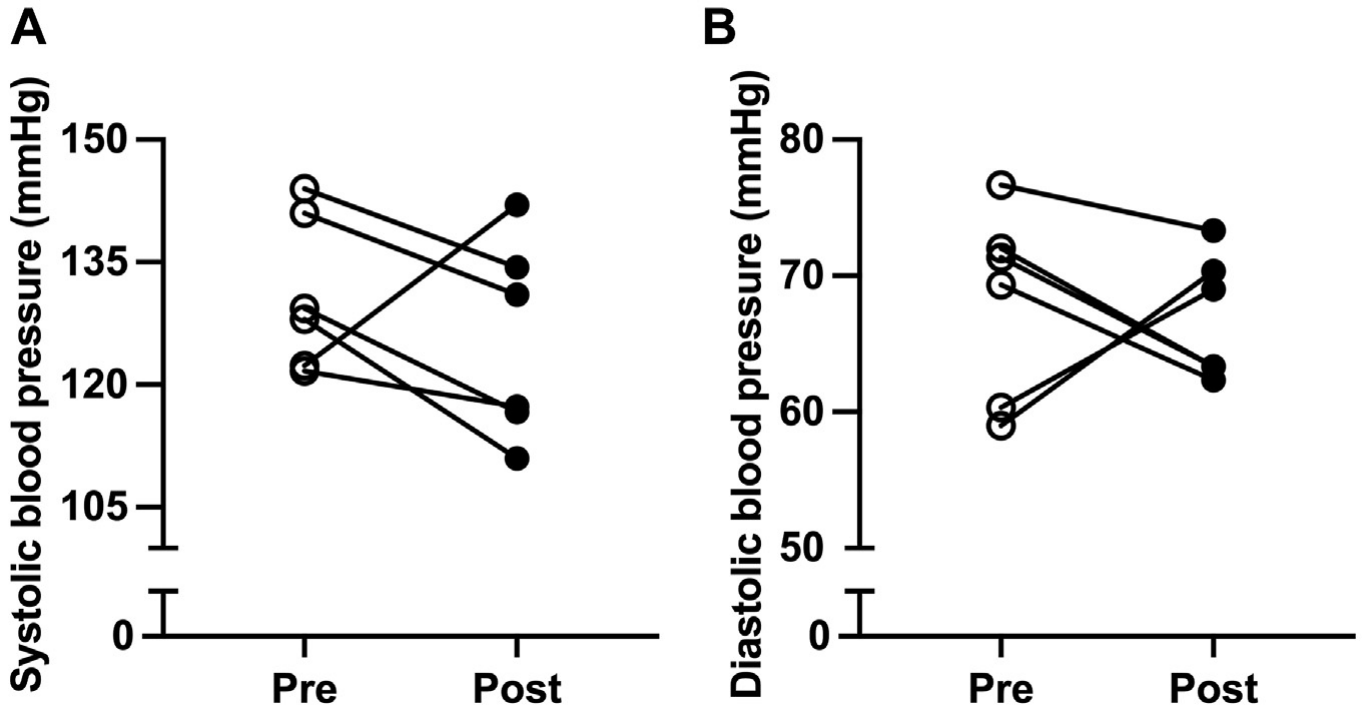
Systolic **(A)** and diastolic **(B)** blood pressure, measured in triplicate in the sitting position, before and after completion of the 12-week leg heat therapy (HT) treatment. Data were compared using paired two-sample *t* tests.

The changes from baseline to the 3-month follow-up in physical function are shown in Fig 5. On average, participants displayed an increase in 6-minute walk distance from baseline of +32.2 meters (*P* = .07; 95% confidence interval [CI], −4.595 to 69.24) (Fig 5, *A*), which is clinically meaningful for patients with PAD.^33^ The improvement in walking endurance was coupled with increased calf muscle strength (+4.4 Nm; *P* = .14; 95% CI, −2.154 to 11.15) (Fig 5, *B*) and a decrease in the time required to complete the five-times sit-to-stand test (−1.9 seconds; *P* = .12’ 95% CI, −4.615 to 0.7420) (Fig 5, *C*). Table III displays the scores from the WIQ. The self-reported walking speed scores increased in five of the six participants (+11 points; *P* = .16; 95% CI, −6.539 to 28.71). As shown in Fig 6, five patients displayed a reduction in systolic blood pressure compared with baseline (−5.66 mm Hg; *P* = .33; 95% CI, −19.40 to 8.062) and four patients had a reduction in diastolic pressure (−1.16 mm Hg; *P* = .76; 95% CI, −10.49 to 8.156).

**Table III:** Change from baseline to the 12-week follow-up in the scores of the walking impairment questionnaire (*WIQ*)

	Baseline	12-Week follow-up	Difference in changes	*P* value
Distance score (%)	61.85 ± 32.78	73.11 ± 27.31	11.26 (−6.65 to 29.16)	.167
Speed score (%)	63.59 ± 28.02	73.55 ± 23.42	9.96 (−7.90 to 27.83)	.211
Climbing score (%)	65.97 ± 31.12	68.75 ± 32.99	2.78 (−20.47 to 26.03)	.771
Overall WIQ score (%)	61.94 ± 32.59	73.02 ± 27.31	11.08 (−6.69 to 28.86)	.170

Values are means ± standard deviation or mean (95% confidence interval). Data were compared using paired two-sample *t* tests.

## Discussion

Building on an existing cryotherapy and compression water-circulating system designed for sports recovery, this study aimed to develop a leg HT system tailored for home-based, unsupervised treatment of elderly individuals with symptomatic PAD. The gentle pneumatic inflation of the bladders was designed to press the water-circulating pads firmly against the skin, ensuring efficient heat transfer regardless of limb dimensions. To ensure safety and ease of use, particularly for elderly users, several safety features were incorporated: (1) a single-touch controller unit integrating a heater, water pump, and air pump for simplified operation, (2) temperature sensors in both the water reservoir and leg sleeves, linked to an automatic shut-off controller to prevent overheating, and (3) a built-in timer to automatically shut off the system after 90 minutes of treatment. We successfully developed a portable, user-friendly prototype with these enhanced safety mechanisms to prevent complications such as skin burns. In a preliminary study involving a small sample of patients with symptomatic PAD, we demonstrated that unsupervised, home-based leg HT using the device daily for 12 weeks is feasible and well-tolerated. These promising findings lay the foundation for the development of a clinical-grade leg HT system with the potential to significantly impact the management of symptomatic PAD.

One of the central challenges in managing symptomatic PAD is the lack of accessible, painless, and widely available therapies.^34^ Owing to this limited range of options, an increasing number of patients with PAD are opting for invasive procedures, including stenting or surgical revascularization. The use of peripheral vascular interventions to treat claudication has increased dramatically over the past decade.^35^^,^^36^ However, the long-term cost effectiveness and durability of these revascularization procedures remain poor.^37^^,^^38^ Patients undergoing revascularization for claudication are at risk of worsening functional status and limb loss.^39^ Leg HT offers a nonpharmacological and noninvasive solution that is suitable for home use, practical for elderly patients with limited mobility, and can be combined with other current therapies, such as exercise training and stenting. The findings from the current feasibility study revealed that unsupervised leg HT has the potential to improve lower extremity physical functioning in symptomatic patients. Consistent with earlier findings from a pilot trial using a garment system originally developed for military applications, participants in the current study demonstrated clinically meaningful improvements in the 6-minute walk distance.^20^ Although the increase from baseline (+32.2 m) was not statistically significant (*P* = .07), it exceeds the threshold for what is considered a large improvement in walking ability for patients with PAD. A recent analysis of 777 participants with PAD revealed that approximately 8- and 20-meter increases in the 6-minute walk distance represent small and large improvements in walking ability, respectively.^33^ In addition to the gains in walking performance, participants also exhibited positive changes in other functional tests, such as calf muscle strength and the time required to complete five chair stands. Collectively, these pilot data underscore the potential of leg HT to improve physical function in PAD and highlight the need for larger, adequately powered trials to establish its efficacy definitively.

This study represents a crucial first step in the development of a leg HT device, and it also highlights the need for significant engineering refinements before a commercial-grade product can be launched. Although all six participants successfully completed the intervention and showed improvements in lower extremity physical function, patient feedback, including usability scores, revealed several design flaws in the prototype units. Notably, the device was found to be unnecessarily large and heavy when filled with water, making it challenging to transport within the home. Some participants also reported water leakage from the reservoir. Additionally, the time required to heat the water to the desired temperature—approximately 40 minutes—was considered too long, leading to extended preparation times. The proposed treatment regimen, based on previous studies,^20^^,^^40^ was perceived as demanding and raised concerns about long-term adherence in a clinical setting. Future efforts should focus on refining the device to improve portability, addressing the heating time, and developing more user-friendly treatment protocols. Additionally, it will be important to develop methods to track compliance with the treatment regimen, especially given the unsupervised, home-based nature of this therapy.

## Conclusions

Repeated exposure to heat stress has been shown to trigger a range of physiological adaptations that could benefit patients with PAD, such as counteracting muscle atrophy,^41^, ^42^, ^43^, ^44^ improving mitochondrial function,^44^^,^^45^ promoting muscle capillary growth,^46^^,^^47^ and enhancing muscle strength.^47^^,^^48^ However, there is currently no practical, accessible method for unsupervised HT for elderly individuals with PAD. This study marks the first step in designing a portable leg heating system specifically for symptomatic patients with PAD, whose quality of life is severely impacted by debilitating leg pain, weakness, atrophy, and consequent mobility impairment.^49^ Our findings indicate that home-based leg heating therapy, using a novel device that combines water-circulating pads with pneumatic compression sleeves, is feasible for elderly patients with PAD. Future engineering and protocol refinements will be essential to further improve portability, simplify application, and encourage long-term adherence to the therapy.

## Author Contributions

Conception and design: JS, PS, RM, BTR

Analysis and interpretation: BR, BTR

Data collection: BR, JS, CB, BTR

Writing the article: BTR

Critical revision of the article: BR, JS, PS, CB, RM, BTR

Final approval of the article: BR, JS, PS, CB, RM, BTR

Statistical analysis: BTR

Obtained funding: JS, PS, BTR

Overall responsibility: BTR

## Funding

Support for this work was provided by the National Institutes of Health Small Business Innovation Research program (1 R43 HL162337-01A1), awarded to Aquilo Sports to develop the prototype heat therapy systems described in this manuscript. B.R. is additionally supported by R01AG073634.

## Disclosures

J.P.S. is the vice president and P.A.S. is the president of Aquilo Sports, LLC. C.B. previously held the role of Lead Engineer at Aquilo Sports, LLC.
